# Regulatory effects of chicken TRIM25 on the replication of ALV-A and the MDA5-mediated type I interferon response

**DOI:** 10.1186/s13567-020-00870-1

**Published:** 2020-12-09

**Authors:** Jin-run Zhou, Jun-hong Liu, Hong-mei Li, Yue Zhao, Ziqiang Cheng, Yan-meng Hou, Hui-jun Guo

**Affiliations:** 1 Shandong Provincial Key Laboratory of Animal Biotechnology and Disease Control and Prevention, Tai’an, 271018 China; 2 College of Animal Science and Veterinary Medicine, Shandong Agricultural University, Tai’an, 271018 China

**Keywords:** chicken TRIM25, subgroup A of avian leukosis virus, antiviral bioactivities, type I interferon response

## Abstract

This study focuses on the immunoregulatory effects of chicken TRIM25 on the replication of subgroup A of avian leukosis virus (ALV-A) and the MDA5-mediated type I interferon response. The ALV-A-SDAU09C1 strain was inoculated into DF1 cells and 1-day-old SPF chickens, and the expression of TRIM25 was detected at different time points after inoculation. A recombinant overexpression plasmid containing the chicken TRIM25 gene (TRIM25-GFP) was constructed and transfected into DF1 cells to analyse the effects of the overexpression of chicken TRIM25 on the replication of ALV-A and the expression of MDA5, MAVS and IFN-β. A small interfering RNA targeting chicken TRIM25 (TRIM25-siRNA) was prepared and transfected into DF1 cells to assess the effects of the knockdown of chicken TRIM25 on the replication of ALV-A and the expression of MDA5, MAVS and IFN-β. The results showed that chicken TRIM25 was significantly upregulated at all time points both in ALV-A-infected cells and in ALV-A-infected chickens. Overexpression of chicken TRIM25 in DF1 cells dramatically decreased the antigenic titres of ALV-A in the cell supernatant and upregulated the relative expression of MDA5, MAVS and IFN-β induced by ALV-A or by poly(I:C); in contrast, knockdown of chicken TRIM25 significantly increased the antigenic titres of ALV-A and downregulated the relative expression of MDA5, MAVS and IFN-β. It can be concluded that chicken TRIM25 can inhibit the replication of ALV-A and upregulate the MDA5 receptor-mediated type I interferon response in chickens. This study can help improve the understanding of the antiviral activities of chicken TRIM25 and enrich the knowledge of antiviral responses in chickens.

## Introduction

Tripartite motif 25 (TRIM25) is a tripartite motif (TRIM) protein family member and is characterized by a conserved structural domain at the N terminus, including a catalytic RING domain, two B-box domains, and a coiled-coil dimerization domain [1, 2]. In addition, TRIM25 has a C-terminal SPRY domain that binds to target proteins, such as the RIG-I receptor [3, 4].

TRIM25 was first reported in 1993 as an oestrogen-responsive finger protein (EFP) that regulates the oestrogen response [5]. Later, it was also found that the protein can mediate RNA virus infection and induce the antiviral response by ubiquitinating intracellular effectors [6, 7]. TRIM25 has been confirmed to be involved not only in the immune response against many RNA viruses [8] but also in the occurrence of tumours in humans, as an important innate immune molecule [9]. It is becoming clear that TRIM25 has a dual role in the regulation of type I interferons (IFNs), such as IFN-α/β; that is, TRIM25 positively regulates IFNs by the K63-linked ubiquitination of RIG-I and negatively regulates IFNs by the K48-linked ubiquitination of MAVS, and it can thus inhibit or enhance the replication of some RNA viruses [10, 11].

In recent years, many animal TRIM25 genes have been cloned, and their molecular structures and antiviral effects have been reported [12, 13]. The chicken TRIM25 gene was cloned in 2015, and it was confirmed that its protein was widely expressed in chickens, especially in immune organs [14, 15]. However, due to the absence of RIG-I in chickens [16, 17], little is known about the immunoregulatory effects of chicken TRIM25 on the replication of RNA viruses and type I IFN responses. In 2015, it was reported that stimulator of IFN genes (STING), as an important regulator of chicken innate immune signalling, might be involved in the MDA5 signalling pathway in chicken cells [18]. In this study, it was found that chicken TRIM25 can inhibit the replication of subgroup A avian leukosis virus (ALV-A) and upregulate the MDA5-mediated IFN-β response in chicken cells.

## Materials and methods

### Cells, virus strain and reagents

DF1 cells, a chicken embryonic fibroblast cell line in which exogenous ALV can replicate, were a gift from Professor Zhizhong Cui. The polyclonal antibody (PAb) against chicken TRIM25 was prepared by our laboratory [15]. The ALV-A-SDAU09C1 strain was isolated from imported meat-type grandparent chickens in 2009 and stored at − 80 °C until use [19]. The 50% tissue culture infective dose (TCID_50_) of the virus was determined using a limiting dilution assay in a 96-well plate covered with DF1 cells according to the Reed–Muench method. The positive cells were identified using an indirect immunofluorescence assay (IFA) mediated by a monoclonal antibody (MAb) against ALV-A. The pEGFP-C1 vector containing the green fluorescent protein gene was purchased from Beijing Tiangen Biotechnology Co., Ltd., and a small interfering RNA (siRNA) was synthesized by Shanghai GenePharma Biotechnology Co., Ltd.

Polyinosinic-polycytidylic acid (poly(I:C)) sodium salt (number: P1530) was purchased from Sigma, dissolved at 5 mg/mL with sterile PBS and stored at − 20 °C. Before transfection, poly(I:C) was diluted to 1 mg/mL and mixed with Lipofectamine 3000 reagent in a 200 µL reaction system with Opti-MEM according to the instructions. Finally, transfection medium containing 2 μg/mL poly(I:C) was added to prepared DF1 cells.

### Viral infection

In vitro, DF1 cells were inoculated with 10^2^ TCID_50_ of the ALV-A-SDAU09C1 strain, and the inoculum was discarded at 2 h post inoculation (hpi); fresh maintenance medium was added onto the infected cells. The cell samples were collected at 0, 2, 6, 12, 24, 72, 120 and 168 hpi. RNA was extracted from the cells to detect the mRNA levels of chicken TRIM25 by real-time PCR (RT-PCR). In addition, the total proteins were also extracted from the cells to detect the protein levels of chicken TRIM25 by western blot mediated with the PAb against chicken TRIM25.

In vivo, thirty 1-day-old SPF chickens (White Leghorn) were intraperitoneally inoculated with 10^4^ TCID_50_ of the ALV-A-SDAU09C1 strain as the ALV-A infected group; in addition, thirty 1-day-old SPF chickens were intraperitoneally inoculated with DMEM as the control group. A total of 0.5 mL of solution was injected into each chicken. Blood samples were collected from all the experimental chickens, and the antigenic titres of ALV-A in the plasma samples were detected by ELISA. Six chickens per group were randomly killed at 1, 2, 3, and 4 weeks post inoculation (wpi), and RNA was extracted from the spleen, liver, kidney and lung of the sacrificed chickens to detect the expression of chicken TRIM25 mRNA by RT-PCR.

### Amplification of the chicken TRIM25 gene

The chicken TRIM25 gene containing the open-reading frame (ORF) was amplified from the spleen of chickens according to a published method [15]. The primers (forward: 5′-CCCGAATTCTTGCAACAACCAGGAAACG-3′; reverse: 5′-CCCGCGGCCGCTCAGTGATGATGATGATGATGCCTGCTACGGCGGTGACCC-3′) were designed according to the chicken TRIM25 gene sequence (NM_001318458.1) published in GenBank and were synthesized by Liuhe Huada Gene Biological Limited Co. Fresh spleen tissues were collected from Hy-Line Brown chickens, RNA was extracted using an RNA Extraction Kit, and reverse transcription was carried out to obtain cDNA encoding chicken TRIM25. The target gene was amplified from the obtained cDNA by polymerase chain reaction (PCR) under the following conditions: initial denaturation at 95 °C for 3 min; 30 cycles of denaturation at 95 °C for 30 s, annealing at 61 °C for 60 s, and extension at 72 °C for 72 s; and a final extension at 72 °C for 10 min. The PCR products were analysed using 1% agarose gel electrophoresis, and then, the PCR products that were recovered using a DNA Gel Recovery Kit were sequenced by Shanghai Biological Engineering Technology Services Limited Co.

### Knockdown of chicken TRIM25 by siRNA

A small interfering RNA (siRNA) targeting chicken TRIM25, named TRIM25-siRNA in Table 1, was designed and synthesized. DF1 cells were seeded at 5 × 10^5^ cells/well in 6-well plates and transfected with 50 nM TRIM25-siRNA mixed with 2 μL of Lipofectamine 3000 (Invitrogen) according to the manufacturer’s instructions. The knockdown effect of TRIM25-siRNA on TRIM25 in DF1 cells was evaluated at 24 h post transfection by real-time RT-PCR and by western blot conducted with the PAb against chicken TRIM25, and the efficiency was determined as the ratio of TRIM25 expression in the knockdown samples relative to that in the negative control samples.

**Table 1:** **TRIM25-SiRNA and control SiRNA sequences used in this study**

Purpose	Name	Sequence (5′ to3′)	Accession no
Knockdown	TRIM25-SiRNA	GCUAACGUCACGCUGGAUUTT	NM_001318458.1
Control	NC-SiRNA	UUCUCCGAACGUGUCACGUTT	

### Construction of an overexpression plasmid containing chicken TRIM25

After the chicken TRIM25 gene containing the complete ORF fragment with a size of 1881 bp was successfully amplified, the recombinant overexpression plasmid named TRIM25-GFP was constructed using the cloning vector pMD18-T and the fluorescent expression vector pEGFP-C1. The TRIM25-GFP plasmid and blank GFP plasmid were transfected into DF1 cells with 2 μL of Lipofectamine 3000 (Invitrogen) according to the manufacturer’s instructions, and the expression efficiency of the TRIM25-GFP plasmid in DF1 cells was evaluated by the real-time RT-PCR method and Western blot mediated with the PAb against chicken TRIM25 at 24 h post transfection; simultaneously, the fluorescence was observed using fluorescence microscopy.

### Overexpression and knockdown of chicken TRIM25 regulation of ALV-A replication

The TRIM25-GFP plasmid and TRIM25-siRNA were transfected into DF1 cells; the GFP plasmid and NC-siRNA were used as controls. At 4 h post transfection, the DF1 cells were inoculated with 10^2^ TCID_50_ of the ALV-SDAU09C1 strain; the inoculum was discarded, and new culture medium was added to the cells, which were then cultured for 7 days at 37 °C. Cell supernatant samples were collected at 6 hpi, 12 hpi, 24 hpi, 48 hpi, 96 hpi, and 144 hpi, and the antigenic titres of ALV-A were detected by ELISA using an IDEXX ALV P27 Antigen Kit (IDEXX USA Inc., Beijing, China). Each sample was tested in triplicate.

### Overexpression and knockdown of chicken TRIM25 regulation of IFN-β responses

The TRIM25-GFP plasmid, TRIM25-siRNA, GFP plasmid and NC-siRNA were transfected into DF1 cells with Lipofectamine 3000 reagent. After 4 h, 2 μg/mL poly(I:C) was transfected or 10^2^ TCID_50_ of the ALV-SDAU09C1 strain was inoculated into DF1 cells to activate type I interferon responses according to the published method [18]. Cell samples were collected at 120 h (5 days) post inoculation (hpi). The mRNA transcript levels of chicken TRIM25, MDA5, MAVS, IFN-β and GAPDH in the cells were detected by real-time RT-PCR, and their relative expression levels were calculated. Each sample was tested in triplicate.

### Detection of ALV-A antigenic titres by ELISA

To determine the titres of ALV-A in the cell supernatant samples or in the plasma samples, the P27 antigen of ALV-A was detected using an ALV P27 antigen ELISA kit (IDEXX USA Inc., Beijing, China) as described [20]. In brief, 1 mL of the dilution fluid from the kit was added to the samples, and the samples were subjected to three freeze–thaw cycles. After sedimentation, the supernatants of the samples were evaluated for ALV P27 antigens in accordance with the manufacturer’s protocol. The antigenic titres of ALV-A were determined by calculating the sample-to-positive control (S/P) ratio using the formula [(mean of sample optical density)-(mean of negative control optical density)]/[(mean of positive control optical density)-(mean of negative control optical density)]. Each sample was tested in triplicate.

### IFA for detecting ALV-A in DF-1 cells

The TRIM25-GFP plasmid and TRIM25-siRNA were transfected into DF1 cells, and the GFP plasmid and NC-siRNA were used as negative controls. Fresh medium was added to DF1 cells at 6 h post transfection to maintain the culture for 24 h; then, 10^2^ TCID_50_ of the ALV-A strain was inoculated into DF1 cells, the inoculum was discarded at 2 hpi, and DF1 cells were cultured with 1% serum medium for 72 h, washed with PBS and fixed with 4% paraformaldehyde for 20 min. Anti-ALV-A antibody (1:100) was incubated for 1 h, CY3-labelled secondary antibody (1:1000, Beyotime) was incubated at 37 °C for 1 h, and the cells were washed with PBS, then incubated with DAPI for 5 min, and observed under a fluorescence microscope after washing with PBS.

### Real-time RT-PCR

Real-time RT-PCR using SYBR Green was performed on a Roche LightCycler 96 to detect the transcript levels of chicken TRIM25, MDA5, MAVS, IFN-β and GAPDH using a Rotor-Gene SYBR Green PCR Kit (QIAGEN, Valencia, CA) and the corresponding primers listed in Table 2. The reaction mixture, which contained 2× UltraSYBR Mixture, 100 nM forward primer, 100 nM reverse primer and 1 μL of cDNA, was subjected to the following thermal cycling conditions: 1 cycle of predenaturation at 95 °C for 10 min; 40 cycles of amplification at 95 °C for 15 s and 60 °C for 1 min; and 1 cycle of melting at 95 °C for 15, 60 °C for 1 min and 95 °C for 15 s.

**Table 2:** **Sequences of the primers used in real-time RT-PCR reaction**

Primer name	Sequence (5′ to 3′)		Accession number
	Forward	Reverse	
GAPDH	GGTGGTGCTAAGCGTGTTA	CCCTCCACAATGCCAA	NM_204305
TRIM25	TACAACCACCACCCTCA	GATGCCAATGCCACAG	NM_001318458.1
MDA5	TGAAAGCCTTGCAGATGACTTA	GCTGTTTCAAATCCTCCGTTAC	AB371640.1
MAVS	CACCCACGAGGTCCATGTG	TGCTTCATCTGGGACATCATTG	NM_001012893.1
IFN-β	TCCAGCTCCTTCAGAATACG	TGCGGTCAATCCAGTGTT	NM_001024836

### Statistics analysis

All the data are expressed as the mean ± standard deviation. The intergroup differences were analysed with ANOVA software, followed by Student–Newman–Keuls tests of multiple comparisons. P < 0.05 was considered statistically significant. P < 0.01 was considered highly significant.

## Results

### TRIM25 expression in DF1 cells is dramatically upregulated by ALV-A infection

**Figure 1:**
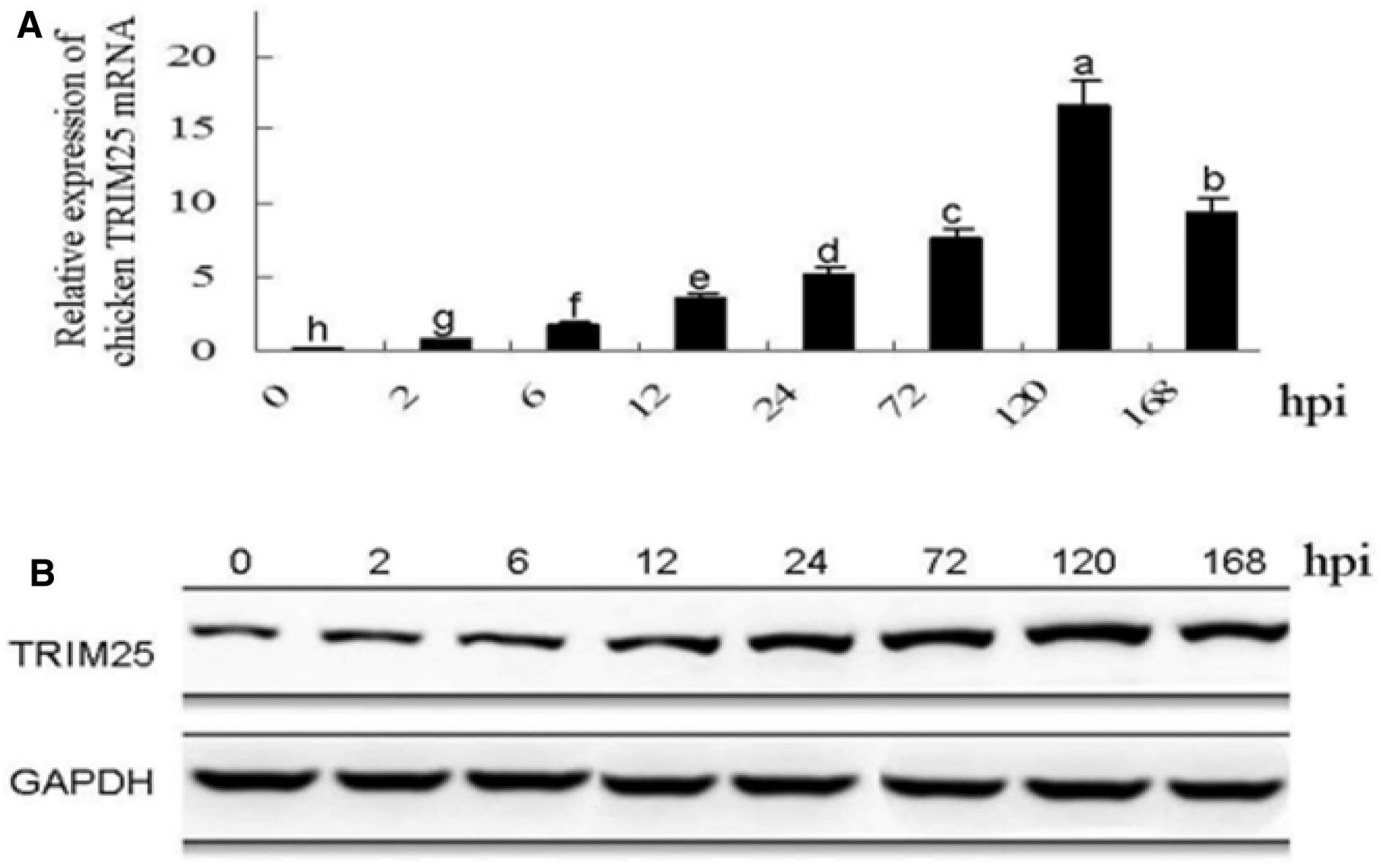
**The mRNA relative expression (A) and the protein expression (B) of chicken TRIM25 in the DF1 cells at different time points after infection with the ALV-A-SDAU09C1 strain.** Different letters on the bars indicate statistically significant differences between the groups (P < 0.05); hpi, hours post inoculation.

As shown in Figure 1A, the mRNA level of chicken TRIM25 in DF1 cells was significantly upregulated from 2 to 168 hpi and reached the highest point at 120 hpi. The western blot results in Figure 1B further confirmed this finding. These results suggest that chicken TRIM25 might be involved in the cell responses to ALV-A infection.

### TRIM25 expression in chickens is significantly upregulated by ALV-A infection

**Figure 2:**
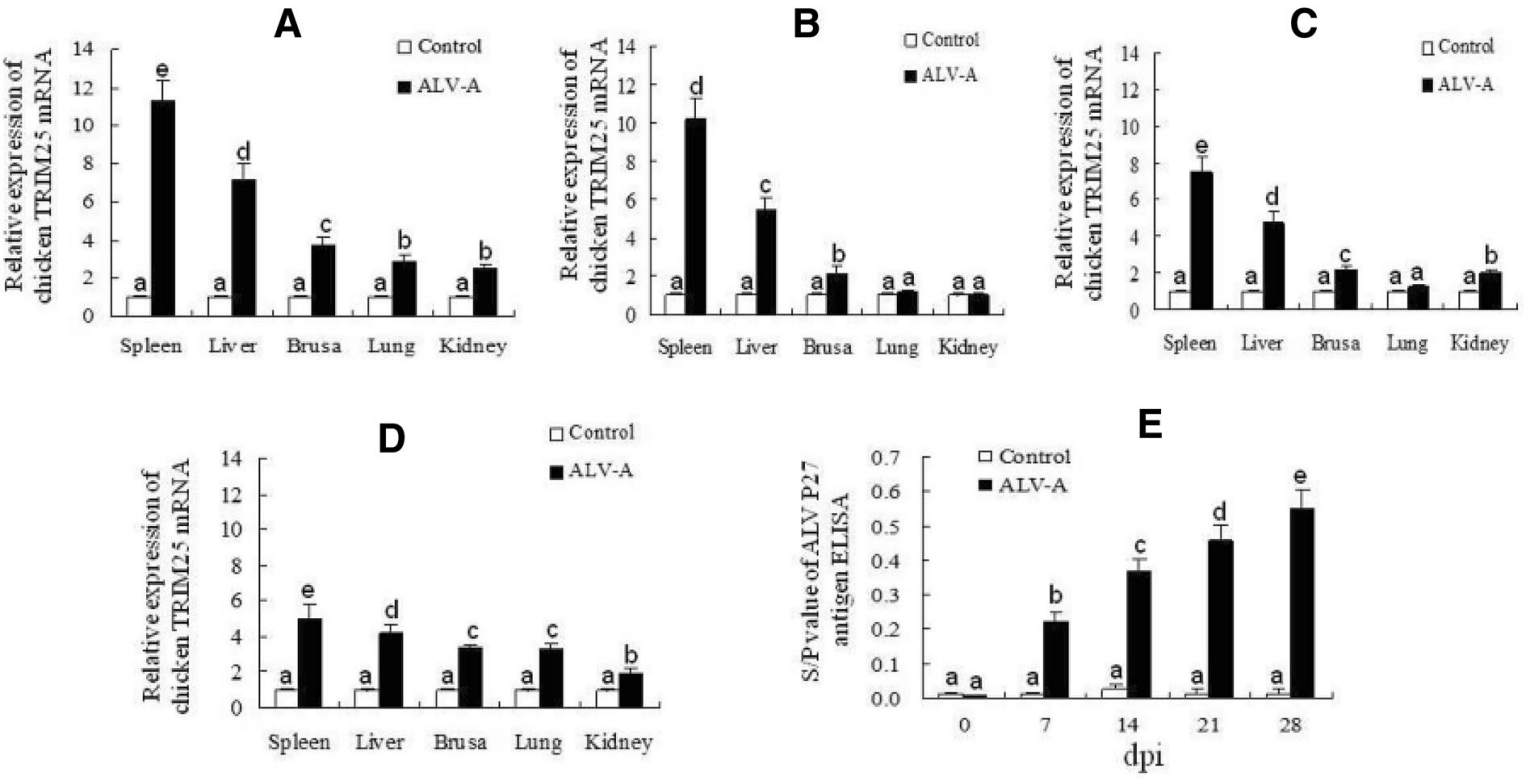
**The relative expression of TRIM25 mRNA in spleen, liver, bursa, lung and kidney of chickens at 7 dpi (A), 14 dpi (B), 21 dpi (C), 28 dpi (D) and the antigen titers of ALV-A in the blood (E).** Different letters on the bars indicate statistically significant differences (P < 0.05) and the same letter indicates no significant difference (P > 0.05) between the groups; dpi, days post inoculation.

To further confirm the expression of TRIM25 after ALV-A infection in vivo, 1-day-old SPF chickens were infected with the ALV-A strain, and the mRNA expression of TRIM25 in five organs, the spleen, liver, bursa, lung and kidney, was detected at 7 dpi, 14 dpi, 21 dpi and 28 dpi. The results in Figure 2A–D show that the relative mRNA expression of TRIM25 in these five organs of the infected chickens was increased by different ratios compared with that in these five organs of the control chickens, especially in the spleens and livers, as the relative expression was increased by 4 to 10-fold in the spleens and by 3.5 to 6-fold in the livers. Simultaneously, it was also found that the expression of TRIM25 decreased with increasing ALV-A inoculation time from 7 dpi (Figure 2A) to 28 dpi (Figure 2D); however, the ALV-A antigenic titres in the infected chickens’ blood were clearly increased from 7 to 28 dpi (Figure 2E). These results suggest that TRIM25 is involved in the immune responses against ALV-A infection in chickens and may negatively regulate the replication of ALV-A in chickens.

### Overexpression of chicken TRIM25 can inhibit the replication of ALV-A

**Figure 3:**
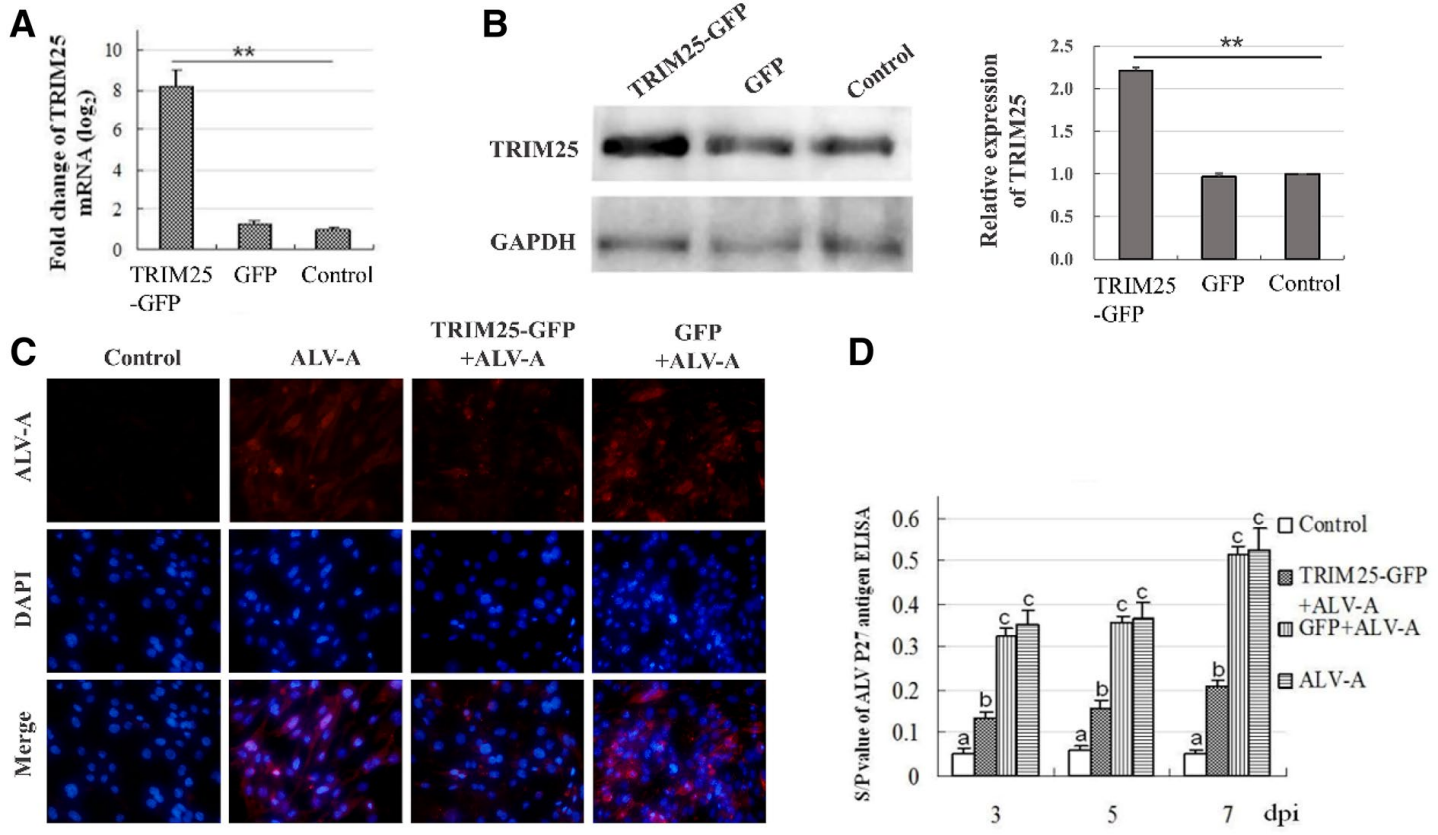
**Overexpression of chicken TRIM25 can inhibit the replication of ALV-A.** The expression of chicken TRIM25 in the DF1 cells transfected with the TRIM25-GFP plasmid and GFP plasmid were detected by QPCR (**A**) and western blot (**B**) (the grey signals were analyzed using Image J software and the relative ratios were statistically calculated, **, P < 0.01). The intracellular proliferation of ALV-A after TRIM25-GFP transfection was detected by immunofluorescence at 3 dpi (**C**). The effects of TRIM25-GFP on the antigen titers of ALV-A in DF1 cells at different days post inoculation with the ALV-A-SDAU09C1 strain were detected by ELISA (**D**). Different letters on the bars indicate statistically significant differences (P < 0.05) and the same letter indicates no significant difference (P > 0.05) between the groups; dpi, days post inoculation.

To analyse the effects of chicken TRIM25 on the replication of ALV-A, a new recombinant plasmid expressing the chicken TRIM25 ORF fragment gene (TRIM25-GFP) was designed and constructed with the fluorescent expression vector pEGFP-C1. The results show that the chicken TRIM25 gene containing a complete ORF fragment with a size of 1881 bp was successfully amplified, and its cloning vector (pMD18-T) and expression vector containing the EGFP gene (TRIM25-GFP) were also successfully prepared (data not shown). The TRIM25-GFP plasmid and GFP plasmid were transfected into DF-1 cells at a concentration of 1 μg/mL, and green fluorescence specifically occurred in the transfected cells at 24 h post transfection (hpt) and was clearly observed with fluorescence microscopy at 72 hpt (data not shown). The mRNA and protein expression levels of chicken TRIM25 were detected by QPCR and western blot, and the results showed that TRIM25 mRNA levels in the TRIM25-GFP-transfected cells were significantly higher than those in the GFP-transfected cells and in the blank control cells (Figure 3A). The western blotting results further confirmed this finding (Figure 3B).

After inoculation with the ALV-A-SDAU09C1 strain, DF1 cells were examined for the presence of ALV-A using IFA at 3 dpi. The results showed that ALV-A levels in the TRIM25-GFP-transfected cells were significantly lower than those in the GFP transfected cells and ALV-A positive control cells (Figure 3C). The antigen titres of ALV-A in DF1 cell supernatants were detected by ELISA at 3, 5 and 7 dpi. The results in Figure 3D show that the ALV-A titres in the TRIM25-GFP-transfected cells were significantly lower than those in the GFP-transfected cells and those in the ALV-A positive control cells at all time points; furthermore, the decrease ratios were more than 50%, although they were significantly higher than those in the uninfected cells. These results suggest that overexpression of chicken TRIM25 in DF1 cells can clearly inhibit the replication of ALV-A.

### Knockdown of chicken TRIM25 can promote the replication of ALV-A

**Figure 4:**
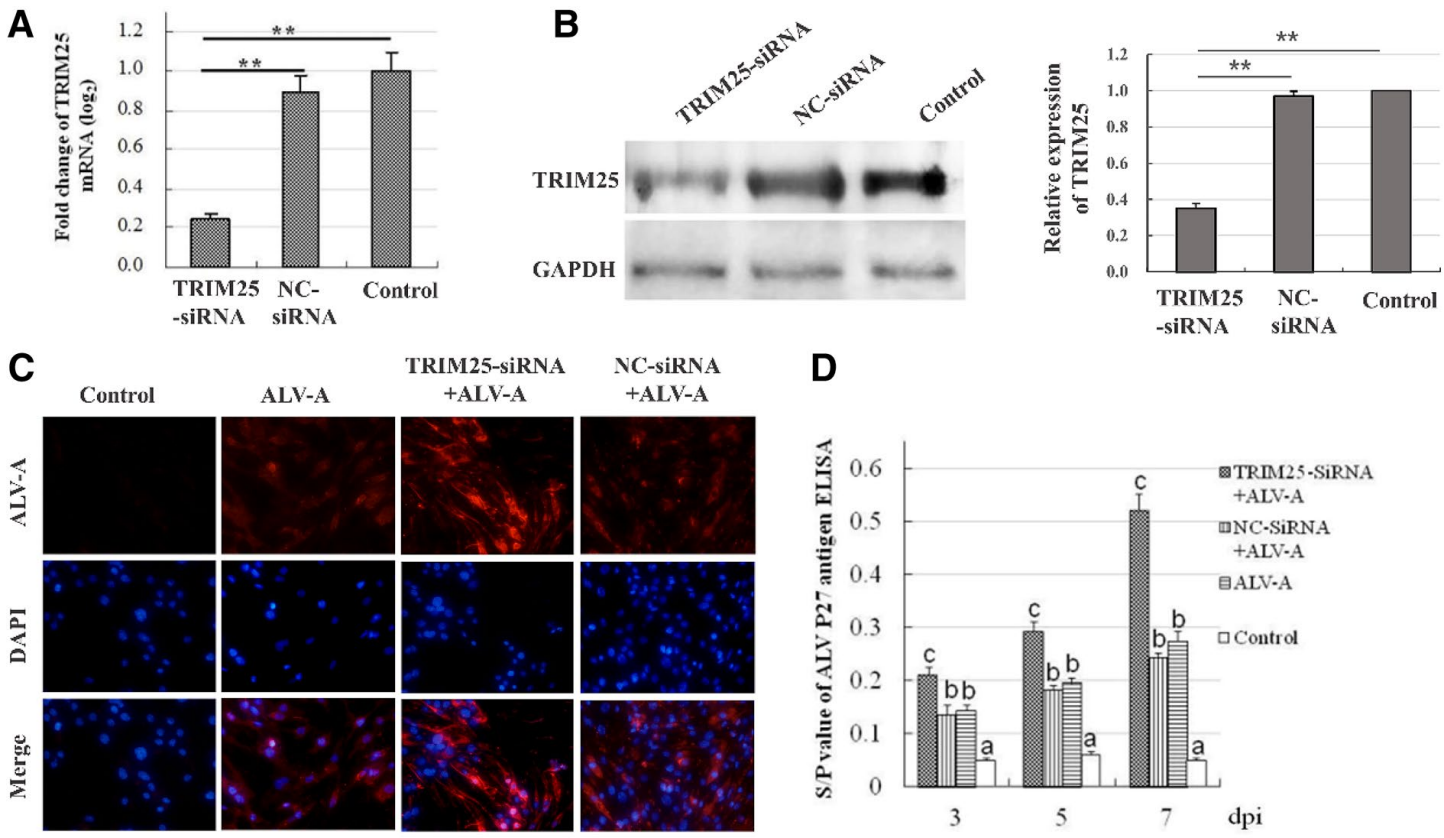
**Knockdown of chicken TRIM25 can promote the replication of ALV-A.** The expression of chicken TRIM25 in the DF1 cells transfected with the TRIM25-siRNA and NC-siRNA were detected by QPCR (**A**) and western blot (**B**) (the grey signals were analyzed using Image J software and the relative ratios were statistically calculated, **, P < 0.01). The intracellular proliferation of ALV-A virus after TRIM25-siRNA transfection was detected by immunofluorescence at 3 dpi (**C**). The effects of TRIM25-siRNA on the antigen titers of ALV-A in DF1 cells at different days post inoculation with the ALV-A-SDAU09C1 strain were detected by ELISA (**D**). Different letters on the bars indicate statistically significant differences (P < 0.05) and the same letter indicates no significant difference (P > 0.05) between the groups; dpi, days post inoculation.

Based on the principle of siRNA, an siRNA construct targeting chicken TRIM25, named TRIM25-siRNA, and a negative control siRNA construct, named NC-siRNA, were designed, as shown in Table 1. These plasmids were transfected into DF1 cells at a dose of 50 nM, and the expression of TRIM25 mRNA and protein was detected by QPCR at 24 hpt and by western blot at 72 hpt, respectively. The results show that TRIM25 mRNA levels in the TRIM25-siRNA-transfected cells were significantly decreased compared with those in the NC-siRNA-transfected cells and in the control cells (Figure 4A). The western blotting results also showed that TRIM25 protein expression in the TRIM25-siRNA-transfected cells was significantly lower than that in the NC-siRNA-transfected cells and that in the control cells (Figure 4B). These results suggest that the designed siRNA can significantly decrease the expression of chicken TRIM25 in DF1 cells and that the knockdown efficiency can reach more than 60%.

After inoculation with the ALV-A-SDAU09C1 strain, ALV-A in DF1 cells was detected using IFA at 3 dpi. The results showed that ALV-A levels in the TRIM25-siRNA transfected cells were significantly higher than those in the NC-siRNA transfected cells and those in the ALV-A positive control cells (Figure 4C). The results in Figure 4D show that the antigen titres of ALV-A in the TRIM25-siRNA-transfected cells were significantly higher than those in the NC-siRNA-transfected cells and those in the ALV-A positive control cells at 3, 5, and 7 days post-inoculation; furthermore, the increase ratios reached 40% ~ 104%. These results suggest that knockdown of chicken TRIM25 in DF1 cells can promote the replication of ALV-A.

### Overexpression of chicken TRIM25 can upregulate the IFN-β response induced by ALV-A infection, but knockdown of chicken TRIM25 can downregulate this response

**Figure 5:**
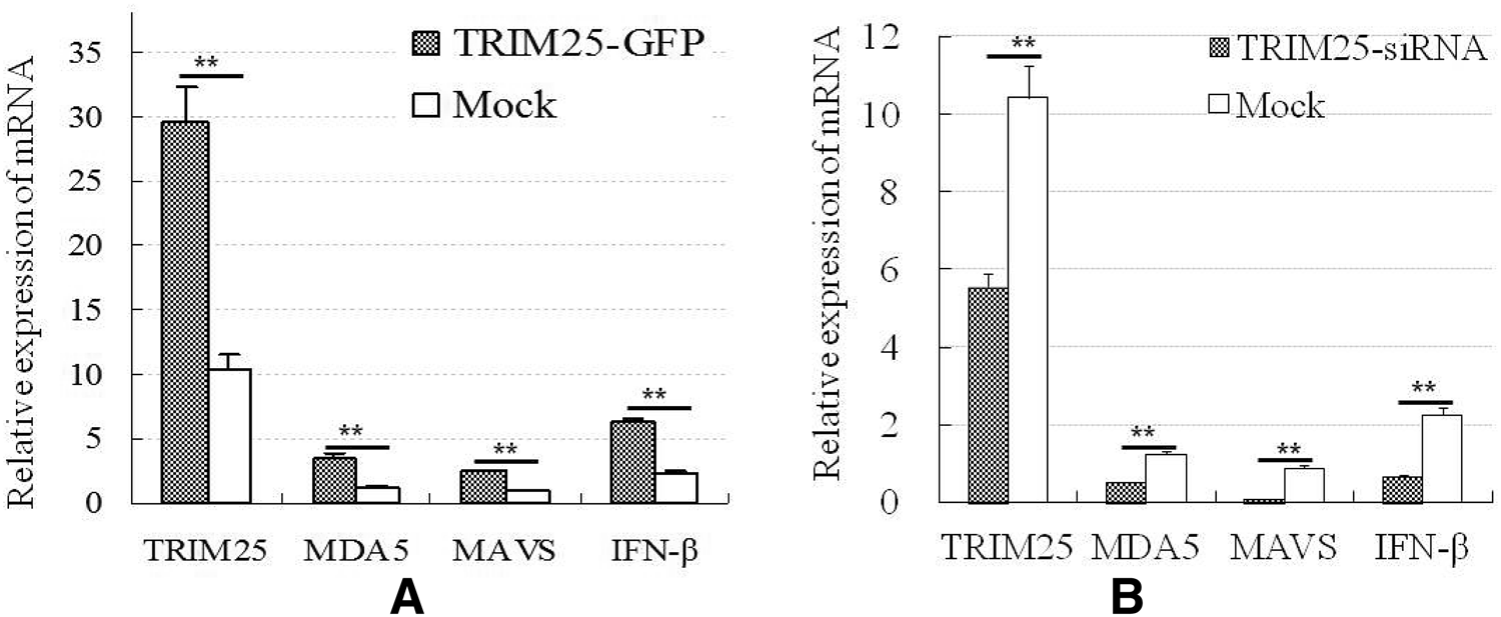
Effects of overexpression (A) and knockdown (B) of chicken TRIM25 on the mRNA transcript levels of MDA5, MAVS and IFN-β induced by ALV-A infection in DF1 cells (*, P < 0.05; **, P < 0.01). The TRIM25-GFP plasmid, TRIM25-siRNA, GFP plasmid (Mock) and NC-siRNA (Mock) were transfected into DF1 cells, respectively; and 10^2^ TCID_50_ of the ALV-SDAU09C1 strain was inoculated at 4 h post transfection, then the cell samples were collected for detection at 120 h post inoculation.

To analyse the effects of chicken TRIM25 on the type I interferon response induced by ALV-A infection, the TRIM25-GFP plasmid and TRIM25-siRNA were transfected into DF1 cells, and the cells were inoculated with the ALV-A-SDAU09C1 strain. The relative expression levels of TRIM25, MDA5, MAVS and IFN-β were detected at 5 dpi. The results in Figure 5 show that the mRNA expression of TRIM25 in the TRIM25-GFP-transfected cells was greatly increased and that the expression of MDA5, MAVS and IFN-β was also significantly increased after ALV-A infection compared with that after mock infection (Figure 5A). In contrast, the mrna level of TRIM25 in the TRIM25-siRNA-transfected cells was decreased, and the expression of MDA5, MAVS and IFN-β was significantly decreased after ALV-A infection (Figure 5B). These results suggest that chicken TRIM25 can upregulate the IFN-β response induced by ALV-A infection.

### Chicken TRIM25 can upregulate the MDA-mediated type I interferon response induced by poly(I:C)

**Figure 6:**
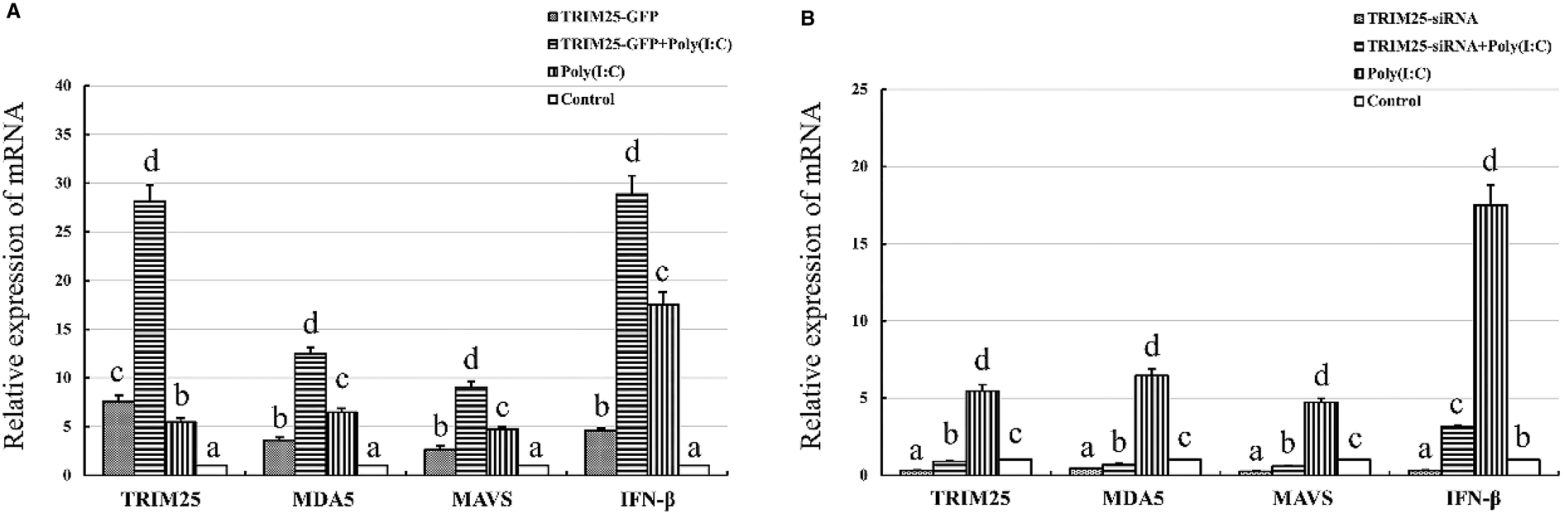
**Effects of overexpression (A) and knockdown (B) of chicken TRIM25 on mRNA expression ofMDA5, MAVS and IFN-β induced by poly(I:C) in DF1 cells.** Different letters on the bars indicate statistically significant differences (P < 0.05) and the same letter indicates no significant difference (P > 0.05) between the groups. The TRIM25-GFP plasmid, TRIM25-siRNA, GFP plasmid and NC-siRNA were transfected into DF1 cells, respectively; and 2 μg/mL poly(I:C) were transfected at 4 h post-first-transfection, then the cell samples were collected for detection at 120 h post-second-transfection.

To further elucidate the signalling mechanism by which chicken TRIM25 affects the IFN-β response induced by ALV-A infection, poly(I:C), a simplified model of ALV-A strain infection, was co-transfected with the TRIM25-GFP plasmid or TRIM25-siRNA into DF1 cells, and the expression of TRIM25, MDA5, MAVS and IFN-β was detected. The results in Figure 6 show that the relative mRNA expression of these molecules was greatly increased in the TRIM25-GFP-transfected cells (Figure 6A) and dramatically decreased in the TRIM25-siRNA-transfected cells (Figure 6B) compared with that in the respective control cells. These results suggest that chicken TRIM25 can also upregulate the MDA5-mediated type I interferon response induced by poly(I:C).

## Discussion

ALV, an oncogenic retrovirus, can cause immunosuppression, decreased production performance, tumours and even death in chickens [20–23]. ALV is divided into 11 subgroups from A to K according to the host range, antibody neutralization and receptor interference studies, and seven of these subgroups, namely, ALV-A/B/C/D/E/J/K, can infect chickens [24–27]. Except for the ALV-E subgroup, the other six subgroups are exogenous ALVs, which can cause severe pathogenicity in chickens [28–30]. ALV-E can interfere with the detection of exogenous ALVs because its gene was inserted into the chicken genome early in its evolution, but its pathogenicity is weak [24, 31]. DF1 cells, a chicken fibroblast cell line with resistance to endogenous ALV [32, 33], have been widely applied to the isolation or detection of exogenous ALVs [34–36]. In this study, we assessed the effect of chicken TRIM25 on the replication of ALV-A in cells.

In this study, DF1 cells were inoculated with the ALV-A-SDAU09C1 strain, which was isolated from imported ancestral breeding broiler chickens in 2009 [19] and was reported to replicate in DF1 cells and to be pathogenic in chickens [19, 20, 37]. The expression of chicken TRIM25 at both the mRNA and protein levels was significantly increased, which suggests that the antiviral responses mediated by TRIM25 were induced. To further confirm this finding, the ALV-A-SDAU09C1 strain was inoculated into 1-day-old SPF chickens, and the relative expression of TRIM25 in the spleen, liver, bursa, lung and kidney was detected. The results showed that the expression of TRIM25 mRNA in these tissues was significantly upregulated after ALV-A infection, especially in the spleen, liver and bursa. In the past, it has been confirmed that TRIM25 is expressed in almost all chicken tissues and organs and is expressed at relatively high levels in immune organs, such as the spleen, thymus, and bursa; moreover, TRIM25 can be clearly upregulated 2 days after infection with Newcastle disease virus (NDV) [14, 15], suggesting that TRIM25 might participate in some antiviral responses in these organs as an important component. In addition to that in immune organs, the expression of TRIM25 in the liver and kidney was also dramatically upregulated, which might result from the tissue tropism of ALV-A because high viral loads and more pathological lesions, such as tumours in these tissues after ALV-A infection, were reported in previous studies [19, 20].

To analyse the effects of chicken TRIM25 on the replication of ALV-A, the complete gene with a size of 1881 bp, which contained an open reading frame (ORF), was amplified from Hy-Line egg-type chicken spleens. The bioinformatics analysis results suggest that the amplified chicken TRIM25 gene has 99.6% homology with the sequences published by Feng (accession No. NM_001318458.1) and contains the typical functional structural domains, such as a RING domain, two B-box domains, a coiled-coil domain and a SPRY domain (unpublished data). Then, this gene was recombined with a eukaryotic expression plasmid containing the green fluorescent protein (GFP) gene to form a new recombinant overexpression plasmid (TRIM25-GFP); meanwhile, a small interfering RNA (TRIM25-siRNA) was prepared to inhibit the expression of endogenous TRIM25 in DF1 cells. The data show that overexpression of TRIM25 can significantly inhibit the replication of ALV-A; in contrast, knockdown of TRIM25 can significantly promote the replication of ALV-A, confirming that chicken TRIM25 has an inhibitory effect on ALV-A replication.

In some previous studies in mammals, TRIM25 could activate the RIG-I protein, which is a receptor that recognizes viral RNA, and induce the production of type I interferons, such as IFN-α/β, which can inhibit the replication of some viruses in cells [2, 38]. Additionally, it was reported that TRIM25 also plays a role in early innate immunity, such as the activation of the MDA5 mitochondrial antiviral signal protein-TRAF6 antiviral axis and the regulation of p53 level and activity [10], and the MDA5 receptor can interact with V proteins of a wide variety of paramyxoviruses to block IFN signal transduction pathways [39]; the inhibition of IFN induction is not limited to species, and the inhibition of MDA5 function is not limited to mammalian cells either [40]. However, there are no reports regarding the mechanism by which chicken TRIM25 inhibits viral replication in chickens due to the absence of the RIG-I gene in chickens. In 2015, Cheng et al. reported that the MDA5 protein, which is another viral RNA recognition receptor, can mediate the type I interferon responses induced by NDV, AIV or poly(I:C) in the absence of the RIG-I receptor [18]. In this study, the MDA5 receptor-mediated type I interferon response was assessed in cells in which chicken TRIM25 was overexpressed or downregulated, and the results showed that all the expression levels of MDA5, MAVS and IFN-β were dramatically upregulated by the overexpression of chicken TRIM25 in response to both incubation with poly(I:C) and inoculation with ALV-A; in contrast, these levels were significantly downregulated by the knockdown of chicken TRIM25. It can be deduced that chicken TRIM25 can increase MDA5-mediated type I interferon responses to produce antiviral factors, such as IFN-α/β, to inhibit ALV-A replication. Notably, further research is needed regarding how chicken TRIM25 activates the MDA5-mediated type I interferon response.

In summary, in ALV-A-infected DF1 cells and ALV-A-infected chickens, the expression of chicken TRIM25 was significantly upregulated, and its overexpression inhibited the replication of ALV-A and upregulated the expression of MDA5, MAVS and IFN-β. In contrast, knockdown of TRIM25 promoted the replication of ALV-A and downregulated the expression of MDA5, MAVS and IFN-β. These findings can help improve the understanding of the antiviral activities of chicken TRIM25 and greatly enrich the knowledge of antiviral responses in chickens.

## Data Availability

All data generated or analysed during this study are included in this published article.
